# Exome-wide study of ankylosing spondylitis demonstrates additional shared genetic background with inflammatory bowel disease

**DOI:** 10.1038/npjgenmed.2016.8

**Published:** 2016-05-04

**Authors:** Philip C Robinson, Paul J Leo, Jennifer J Pointon, Jessica Harris, Katie Cremin, Linda A Bradbury, Simon Stebbings, Andrew A Harrison, Australian Osteoporosis Genetics Consortium, Eugene McCloskey, John Eisman, Graeme Jones, Geoff Nicholson, Richard Eastell, Philip Sambrook (deceased), Richard Prince, Elaine Dennison, Ian Reid, John Wark, Wellcome Trust Case Control Consortium, Management Committee, Peter Donnelly, Ines Barroso, Jenefer M Blackwell, Elvira Bramon, Matthew A Brown, Juan P Casas, Aiden Corvin, Panos Deloukas, Audrey Duncanson, Janusz Jankowski, Hugh S Markus, Christopher G Mathew, Colin NA Palmer, Robert Plomin, Anna Rautanen, Stephen J Sawcer, Richard C Trembath, Ananth C Viswanathan, Nicholas W Wood, Data and Analysis Group, Chris C A Spencer, Gavin Band, Céline Bellenguez, Colin Freeman, Garrett Hellenthal, Eleni Giannoulatou, Matti Pirinen, Richard Pearson, Amy Strange, Zhan Su, Damjan Vukcevic, Peter Donnelly, DNA, Genotyping, Data QC and Informatics Group, Cordelia Langford, Sarah E Hunt, Sarah Edkins, Rhian Gwilliam, Hannah Blackburn, Suzannah J Bumpstead, Serge Dronov, Matthew Gillman, Emma Gray, Naomi Hammond, Alagurevathi Jayakumar, Owen T McCann, Jennifer Liddle, Simon C Potter, Radhi Ravindrarajah, Michelle Ricketts, Matthew Waller, Paul Weston, Sara Widaa, Pamela Whittaker, Ines Barroso, Panos Deloukas, Publications Committee, Christopher G Mathew, Jenefer M Blackwell, Matthew A Brown, Aiden Corvin, Chris CA Spencer, Emma L Duncan, David M Evans, Paul B Wordsworth, Matthew A Brown

**Affiliations:** 1 School of Medicine, Faculty of Medicine and Biomedical Sciences, University of Queensland, Brisbane, QLD, Australia; 2 University of Queensland Diamantina Institute, Translational Research Institute, Princess Alexandra Hospital, Brisbane, QLD, Australia; 3 Department of Rheumatology, Royal Brisbane and Women’s Hospital, Herston, QLD, Australia; 4 Institute of Health and Biomedical Innovation, Queensland University of Technology, Translational Research Institute, Princess Alexandra Hospital Herston, QLD, Australia; 5 National Institute for Health Research (NIHR) Oxford Comprehensive Biomedical Research Centre and Musculoskeletal Biomedical Research Unit, Nuffield Orthopaedic Centre, Oxford, UK; 6 Department of Medicine, University of Otago, Dunedin, New Zealand; 7 Department of Medicine, University of Otago, Wellington, New Zealand; 8 Department of Endocrinology and Diabetes, Royal Brisbane and Women's Hospital, Herston, QLD, Australia; 9 School of Medicine and University of Queensland Centre for Clinical Research, Faculty of Medicine and Biomedical Sciences, University of Queensland, Brisbane, QLD, Australia

## Abstract

Ankylosing spondylitis (AS) is a common chronic immune-mediated arthropathy affecting primarily the spine and pelvis. The condition is strongly associated with HLA-B*27 as well as other human leukocyte antigen variants and at least 47 individual non-MHC-associated variants. However, substantial additional heritability remains as yet unexplained. To identify further genetic variants associated with the disease, we undertook an association study of AS in 5,040 patients and 21,133 healthy controls using the Illumina Exomechip microarray. A novel association achieving genome-wide significance was noted at *CDKAL1*. Suggestive associations were demonstrated with common variants in *FAM118A*, *C7orf72* and *FAM114A1* and with a low-frequency variant in *PNPLA1.* Two of the variants have been previously associated with inflammatory bowel disease (IBD; *CDKAL1* and *C7orf72*). These findings further increase the evidence for the marked similarity of genetic risk factors for IBD and AS, consistent with the two diseases having similar aetiopathogenesis.

## Introduction

Ankylosing spondylitis (AS) is an inflammatory arthropathy with a prevalence of 0.1–0.5% in populations of European or Asian descent. The condition primarily affects the sacroiliac joints and the spine, initially causing pain and reversible stiffness. Subsequent ankylosis of these leads to fixed spinal deformity and increasing disability. The condition also has extra-articular manifestations, most commonly in the eye (acute anterior uveitis), and rarely in the aorta, kidneys and lungs. Inflammatory bowel disease (IBD; either Crohn’s disease or ulcerative colitis) is found in 5–10% of AS patients, and ~60% of AS patients have subclinical ileal inflammation. The disease is known to be highly heritable, and to date at least 47 independent genetic variants have been shown to be associated with AS susceptibility.^1^ These findings have contributed greatly to increased understanding of the pathogenesis of the disease, and also to the development of new treatments.^2^ The genetic variants associated with AS have been discovered using coding variant scans,^3^ genome-wide association studies,^4,5^ candidate gene studies^6–10^ and studies targeting immunologically important regions.^11^ Generally these associations have been with common variants. For example, in the International Genetics of AS Consortium Immunochip study, which identified 37 AS-associated variants, the mean minor allele frequency (MAF) of the key-associated single-nucleotide polymorphism (SNP) was 32%, and only two low-frequency (MAF 1–5%) AS-associated alleles were identified and one rare allele (MAF<1%).^11^ As with many common heritable diseases, a large proportion of the heritability of AS remains unexplained. The vast majority of human genetic variation consists of low-frequency and rare variants with MAF<5%.^12^ Studies performed to date have not addressed such variants extensively due to the design of genome-wide association study SNP microarrays and the sample sizes of the studies not having power to detect association with low-frequency or rare variants.^13^ It is likely that there is a substantial numbers of additional variants in the low-frequency or rare variant range that remain undiscovered.

Exomes are proposed to carry a disproportionately high number of clinically important variants because of their potentially profound effects on the protein function.^14^ Much of the variation found in exomes is also rare.^15,16^ Recent exome-sequencing analyses have demonstrated the huge number of rare and potentially damaging variants present in human exomes, with ~313 genes per person predicted to be adversely affected by exonic variants.^14^

In this study, we sought to identify further AS-associated genetic variants focusing on exons using the Illumina HumanExome Beadchip microarray. In addition to covering common coding variants, this chip has extensive low-frequency and rare variant content, enabling us to perform a relatively low cost survey of the role of such variants in AS (compared with studies utilising whole-exome or -genome sequencing).

## Results

### Participants and SNPs

After participant quality control (QC) there were 4,602 AS cases and 20,164 healthy controls. After SNP QC there were 207,193 SNPs. Principal components analysis was performed with 0–10 eigenvectors; the scree plot of genomic control-1000 statistics for different numbers of eigenvectors is shown in Supplementary Figure 1. Use of one eigenvector produced a genomic control-1000 statistic of 1.045, and use of additional eigenvectors did not reduce the genomic control-1000 statistic further. Therefore, all further analyses used a single eigenvector to control for population stratification. The quantile–quantile plot for the experiment is shown in Supplementary Figure 2.

### Single variant analysis

Assays for 10 non-MHC SNPs previously associated with AS were included on the HumanExome Beadchip, and each one showed at least suggestive association (*P*<5×10^−6^) with AS in the current study (Table 1 and Supplementary Figures 3–13). The associations with SNPs in *ERAP1*, *IL23R*, the intergenic regions chromosomes 2p15 and 21q22, *GPR35* and *IL6R* were confirmed at genome-wide significance level (*P*<5×10^−8^). SNP associations within *ANTXR2*, *FCGR2A, IL1R1* and *NOS2* were confirmed at suggestive levels of significance. At nine of the confirmed loci, association was seen with the primary associated SNP. At *NOS2* the association was with rs2297518, which has been reported previously as a secondary signal at the *NOS2* locus, the primary associated SNP rs2531875^11^ not being included on the HumanExome Beadchip.

Novel common variant association was identified in *CDKAL1* (MAF=0.22, *P*=1.8×10^−8^, OR=1.2) at a genome-wide level of significance (Table 2 and Supplementary Figure 14). The *CDKAL1* SNP rs6908425 has previously been associated with Crohn’s disease,^17,18^ psoriasis^19^ and ulcerative colitis^20^ again with the C allele being the risk variant. rs6908425 is in strong linkage disequilibrium with variants associated with diabetes and body mass index (Supplementary Table 1).^21^

Suggestive associations were seen with common variants in *FAM118A* (*P*=5.9×10^−8^, odds ratio (OR)=1.2), *C7orf72* (*P*=1.9×10^−7^, OR=1.1) and *FAM114A1* (*P*=1.4×10^−6^, OR=1.1), and with a rare variant in patatin-like phospholipase domain containing 1 (*PNPLA1)* (MAF=0.0017, *P*=1.5×10^−6^, OR=2.6). SNPs in *C7orf72*, including the same SNP we have identified as being associated with AS (rs1456896), have been associated with Crohn’s disease.^18^ *FAM118A* variants have previously been associated with bone mineral density.^22^ Strong linkage disequilibrium exists between the bone mineral density-associated SNP (rs136564) and the AS-associated SNP, rs6007594 (*r*^2^=0.03, *D*ʹ=0.92). Functional annotation for the variants is shown in Supplementary Table 2.

After conditioning on the primary association signals there were second, independent signals observed in *ERAP1* and *IL23R* (Table 3 and Supplementary Figure 19). The pattern of association at *ERAP1* is consistent with previous work that describes two independent haplotypes at *ERAP1*, tagged by the functional variants rs30187-T (primary signal) and rs10050860-C (primary unconditioned signal, *P*=3.1×10^−24^, allele C, OR=1.4, 95% confidence interval (CI) 1.27–1.43 and conditioned on rs30187 *P*=9.7×10^−10^, allele C, OR=1.2, 95% CI 1.14–1.30). Moderate linkage disequilibrium was observed between rs30187 and rs10050860 (*r*^2^=0.156 and *D*ʹ=1.00). As previously reported, after conditioning on rs30187, association was confirmed with the neighbouring *ERAP2* gene (rs2549794, *P*=2.8×10^−14^, allele C, OR=1.2, 95% CI 1.16–1.28). This association remains after controlling for both the rs30187 and rs10050860 *ERAP1* variants (rs2549794, *P*=4.7×10^−14^, allele C, OR=1.2, 95% CI 1.16–1.28). No additional associated variants were evident.

At *IL23R*, the intronic variant rs11465804 was slightly more significantly associated than the previously reported rs11209026 nonsynonymous SNP (*P*=1.3×10^−25^ and *P*=4.55×10^−25^, respectively). These two SNPs are in high linkage disequilibrium (*r*^2^=0.89, *D*ʹ=0.97), and conditioning for either SNP controlled for the association at the other SNP, suggesting that the rs11465804 variant tags this previously demonstrated functional SNP.^23,24^ As previously reported, a secondary association is seen at the *IL23R* locus, evident after conditioning on rs11465804 (rs10889677, *P*=9.8×10^−9^, allele A, OR=1.2, 95% CI 1.10–1.20).^3^ No additional associated variants were evident.

An independent signal supported by multiple SNPs was demonstrated after conditioning on the main SNP at the *FAM114A1* locus; these SNPs sit in the *TLR10* gene (Table 3 and Supplementary Figure 22). SNPs in *TLR10* have previously been associated with Crohn’s disease,^25,26^ and the peak-associated SNP in our study, rs1109695, is in strong linkage disequilibrium with the most strongly associated, previously reported, Crohn’s disease *TLR10* SNPs, rs7658893 (*r*^2^=0.64, *D*ʹ=1.0) and rs6841698 (*r*^2^=0.64, *D*ʹ=0.83).

### Rare variant burden tests

Using the SKAT-O test, no genes were significantly associated with AS at either a genome-wide or suggestive level of significance. This was the case for all the sets of variants incorporated into the burden equation including the following: (a) ‘Damaging set’, (b) ‘Polyphen set’ and (c) all variants. No association was identified even after removing the restrictive minor allele filter of 0.05 and including SNPs of all frequencies.

With sample sizes of 4,602 AS cases and 20,164 controls used in this study, the power to detect the association was good for low MAF variants, but low for rare variants (Supplementary Figure 23). Assuming a prevalence of 0.55%, allele risk of 1.5 and alpha of 5×10^−8^, and equal MAF for the disease-causative and genotyped markers, the study had 100% power for MAF=0.05, but only 9% power for MAF=0.01 and close to zero (6.1×10^−5^%) power for the median MAF in this study (2.0×10^−4^).

## Discussion

This study re-demonstrates a number of known AS genetic associations both within and outside the MHC (*ERAP1*, *IL23R*, chromosome 2p15, chromosome 21q22, *KIF21B*-*GPR25*, *GPR35*, *IL6R*, *ANTXR2*, *IL1R1*, *FCGR2A* and *NOS2*). It also describes a common novel AS-associated non-MHC variant that achieved genome-wide significance (*CDKAL1*), and three novel common variants that achieved a suggestive level of significance (*FAM118A*, *C7orf72*, *FAM114A1*). One new suggestive rare variant association in *PNPLA1* was identified in single marker analyses, in addition to low-frequency associations observed with rs11465804 in *IL23R* and rs4349859 in HLA-B*27. However, no rare variant associations were noted using burden tests.

In the current study, we identify three AS-associated variants that have previously been associated with IBD, namely variants in or near *CDKAL1, C7orf72* and *TLR10*. There is a high prevalence of IBD in patients with AS;^27,28^ around 10% of AS patients have clinical IBD and up to 70% have subclinical bowel inflammation demonstrated histologically.^29^ In addition, reactive arthritis, which is a type of spondyloarthritis that can progress to AS, can be triggered by enteric infections such as *Campylobacter*, *Salmonella* and *Shigella*. Strong co-familiality between AS and IBD exists, the sibling recurrence risk ratio for IBD in first-degree relatives of AS probands being 3.0,^30^ not dissimilar to the overall familiality of rheumatoid arthritis.^31^ There is extremely strong correlation between AS and IBD genetic associations, with a 2013 analysis indicating that the two diseases shared at that point 22 SNP associations, of which 21 were concordant (same SNP, same direction of association).^32,33^ Nonetheless, major differences exist between genetic associations of the two diseases pointing to differences in disease-specific aetiopathogenesis; for example, the absence of association of IBD with HLA-B*27, and the absence of association of AS with the major IBD loci *NOD2/CARD15* and *ATG16L1*. The finding of three more concordant genetic associations further strengthens the evidence of shared aetiopathogenesis between these diseases.

Little is known about *FAM114A1*. It may be responsible for the described association, but the corresponding protein NOXP20 contains a predicted caspase recruitment (CARD) domain suggesting that it may be involved in apoptosis.^34^ *CARD9* has previously been associated with AS and NOXP20 may have a similar role. In addition, neighbouring genes include the Toll-like receptors *TLR1* and *TLR6* that are intimately involved in innate immunity and so are strong biological candidates for involvement in AS. The second independent signal at the *FAM114A1* locus was in the toll-like receptor 10 gene (*TLR10)*. This missense variant (rs11096955) is predicted by Polyphen-2 to cause a benign change (isoleucine to leucine), but it may tag other more functional variants. This association implicates this important component of the innate immune system in AS aetiology. Toll-like receptors recognise evolutionary conserved sequences on pathogens and trigger immune responses. TLR10 has been recently identified to induce pro-inflammatory cytokine production and interferon in response to influenza infection.^35^ It has recently been suggested that immunodeficiency to gut organisms may trigger AS; if the association with *TLR10* impairs innate immune responses, this would be consistent with this theory.^36^

*FAM118A* is a protein-coding gene of unknown function that encodes a single-pass transmembrane protein (www.uniprot.org). The AS-associated SNP (rs6007594) is a missense mutation causing an arginine to be replaced by a histidine. This missense change is predicted to be probably damaging with a Polyphen-2 score of 0.999 (sensitivity: 0.14; specificity: 0.99).^37^ Kwan *et al.*^22^ demonstrated that FAM118A is expressed in lymphoblastoid cell lines as well as human osteoblasts, and in both cell types showed major SNP effects on FAM118A expression levels. How this impacts on AS aetiology is not immediately evident and larger studies studying SNPs across this locus will be required to determine the genetic variant(s) responsible for the association observed here.

The *PNPLA1* gene belongs to a family of genes, the members of which have diverse lipolytic and acyltransferase activities. The function of *PNPLA1* itself is not well understood. It is expressed in epidermal keratinocytes, and has a role in glycerophospholipid metabolism in the cutaneous barrier. Variants in *PNPLA1* are associated with the skin disorder ichthyosis.^38^ The rs141744967 variant is a missense polymorphism that causes a change from alanine to valine. The functional effect is not available from the Polyphen-2 server; both amino acids are non-polar but differ in size by 28 Da. Further larger studies will be required to determine whether this gene is definitively associated with AS.

This study has several potential limitations; the major limitation is power. The power to detect rare variants is a function of their allele frequency and their effect size along with the population frequency of disease and the required statistical significance threshold. While the study had excellent power to detect common variant associations, the power to detect rare variants was low. Considering variants with a frequency of 0.01 (1%), population disease frequency of 0.005 (0.5%) at a significance threshold of *P*=5×10^−8^ using the participant numbers in this study, the study only had 80% power to detect variants with an additive relative risk of >1.8; other than human leukocyte antigen (HLA) associations, few such variants have been reported in common diseases. This demonstrates poor power to detect individual associations, and increasing the number of cases would improve this power. Nonetheless, if there are large numbers of rare variant associations contributing to AS, the study should have had good power to detect some of these, assuming its coverage of rare variants was good.

The coverage of the Exomechip microarray of rare variants is far from comprehensive, and this impacts both on the coverage of the study, and its ability to pinpoint genetic associations. Further fine-mapping and functional studies will be required to confirm whether the genes we have implicated at each locus are themselves directly involved in AS, or if the SNP associations observed operate by influences on other genes. Sequencing of whole genomes has demonstrated millions of low frequency and rare variants that are not covered on the chip, for example, in the low-coverage analysis of 1000 genomes 15.5 million variants were identified.^12^ This suggests that comprehensive rare variant microarray studies may not be feasible, although improvements in imputation methods raise the possibility that many rare but not unique variants may be addressable by this approach.^39^

Finally, rare variants do not share linkage disequilibrium with many other surrounding variants to the extent that common variants do. Therefore if identified in a study such as this, a good check of association is manual inspection of the genotype intensity clustering, and, in addition, considering the biological plausibility. However, probes can map to other areas of the genome without our knowledge giving well-clustered intensity plots. Biological plausibility is not necessarily a good measure of a true association as the association may be the first association in a pathway not previously known to be involved in disease aetiology. This makes independent replication studies essential, although it is particularly challenging for low-frequency or rare variants because of the sample size requirements.

This study has re-demonstrated many known AS risk loci, and also identified a novel common variant at a genome-wide level of significance, and four suggestive associations, including one rare variant association. The finding of further concordant associations with *IBD* genes increases the evidence of shared aetiopathogenesis between the diseases and the potentially important role of intestinal dysbiosis in AS.^40,41^ The major overlap between AS and IBD is also supported by another study showing similar genetic variants but differing effect sizes between variants associated with AS and anterior uveitis.^42^ Whether low-frequency and rare genetic variants are major contributors to the aetiopathogenesis of AS remains unclear and will likely require much larger studies with more comprehensive coverage of these variant types to resolve.

## Materials and methods

### Patient cohorts

AS patients of European descent who met the modified New York criteria^43^ from the United Kingdom, Australia and New Zealand were recruited (*n*=5,040). Healthy controls were provided by the following groups (1) 1958 British Birth Cohort (*n*=5,964); (2) GoDarts type 2 diabetes cohort (*n*=1,793); (3) Oxford Biobank (*n*=4,522); (4) Twins UK cohort (*n*=1,189); (5) Anglo-Australasian Osteoporosis Genetics Consortium (*n*=7,665). All patients gave written informed consent and ethical approval was provided by all appropriate institutional review boards.

### Genotyping and quality control

Each cohort was genotyped using the Illumina Infinium HumanExome BeadChip version 1.2. This Illumina microarray has ~240,000 markers, made up of exonic variants, splice variants, stop altering variants, ancestry informative markers and MHC tag SNPs. Genotype calling was completed with zCall.^44^ Each cohort had QC completed separately, assessing missingness by individual (threshold <3%), missingness by genotype (threshold<3%), Hardy-Weinberg equilibrium in controls (Chi-square test threshold *P*=0.01), extreme heterozygosity (threshold>3 standard deviations from mean) and identity by descent threshold of PI_HAT 0.20 was used. After laboratory QC that excluded ~3,000–5,000 SNPs per cohort, 20,714–22,864 SNPs were removed from each set to form a common SNP basis, 1,033 SNPs were removed due to excess missingness, 526 SNPs were removed from control sets due to not being in Hardy–Weinberg equilibrium and 11,711 SNPs were removed due to allele and frequency inconsistencies between the cohorts. 979 subjects were removed due to excess relatedness, 207 were excluded due to extreme heterozygosity and 2 were excluded due to excessive missingness (after SNP QC).

Shared genotyped SNPs between cohorts with MAF>0.05 were then used to perform principal component analysis for ethnicity identification using SHELLFISH (http://www.stats.ox.ac.uk/~davison/software/shellfish/shellfish.php). Unsupervised model-based clustering implemented in R with MCLUST was used to exclude patients deemed to be non-European after plotting with HapMap controls. This model assigns a cluster to each individual based on their principal component analysis values and in consideration to the weighted centre of each cluster and therefore assigns non membership status to those who don’t cluster with core HapMap groups. This analysis identified 219 subjects who were removed due to non-European ethnicity. Supplementary Figures 24 and 25 show the principle component analysis after quality control both with and without the addition of Hapmap Samples.

The genomic control measure (GC or *λ*) was calculated using the designer suggested random set of 5,000 synonymous variants (http://genome.sph.umich.edu/wiki/Exome_Chip_Design#Random_set_of_synonymous_variants_.28as_comparator.29).

### Association analysis

For single variant and low-frequency variant analysis, we followed the procedure used by Peloso *et al.*^45^ For single-variant analysis, we restricted analysis to variants where the frequency was >0.08%, meaning that 20 or more copies of the minor allele were present. We used Plink to perform association analyses with one eigenvector as a covariate for population stratification control. Significance levels used were, genome-wide *P*<5×10^−8^, and suggestive 5×10^−8^*>P*<5×10^−6^.

For low-frequency variant analysis we used the sequence kernel association test–Optimal (SKAT-O) test that computes the SKAT test^46^ and a burden test^47–50^ and then selects the test with the best power.^51^ This was implemented with the ‘skatMeta’ R package. For the low-frequency variant analysis, we used variants with a frequency of <5%. We also only included gene-based tests where there were at least two variants contributing each with MAF >0.08%, thus ensuring there were at least 40 copies of the minor alleles in the test. Three sets of variants were sequentially used as inputs into the SKAT-O test. This is because non-damaging variants can reduce the power to detect associations in burden tests. The sets used were (a) All variants, (b) ‘Polyphen2 set’: Polyphen-2^37^ predicted possibly damaging or probably damaging, and (c) ‘Damaging Set’: Variants causing the following consequences: frameshift substitution, nonframeshift substitution, nonframeshift deletion, nonframeshift insertion, frameshift deletion, frameshift insertion, nonsynonymous single-nucleotide variant, stop-gain single-nucleotide variant, stoploss single-nucleotide variant, missense variant, splice acceptor variant, splice donor variant, splice region variant, initiator codon variant, stop retained variant and incomplete terminal codon variant.

Cluster plots for reported SNPs were checked manually in the case cohort and the 1958 British Birth Cohort. Association of classical alleles was completed with imputed SNP2HLA classical alleles and one principal component by logistic regression in R. The level of significance for this analysis was 1.2×10^−4^, reflecting Bonferroni correction for the 424 HLA alleles that were tested for association. Conditional analyses were completed by adding the allele being conditioned as a covariate to the logistic regression model. Power calculations were performed with the online Genetic Power Calculator.

## List of Websites

Genetic Power Calculator: (http://pngu.mgh.harvard.edu/~purcell/gpc/)

SNAP browser: (http://www.broadinstitute.org/mpg/snap/)

## Figures and Tables

**Table 1:** Existing AS genetic associations and new associations in previously associated loci

*Gene/locus*	*Cs*	*Pos*^a^	*Current SNP*	P*-value*	*Risk/Prot*	*OR, 95% CI*	*RAF case/Con*	*Previously reported SNP*	r^*2*^	Dʹ	*Discovery Study*
*ERAP1*	5	96,244,549	rs30187	3.0×10^−34^	T/C	1.3 (1.28–1.41)	0.40/0.34	rs31087	1.00	1.00	Burton *et al.*^3^
*IL23R*	1	67,702,526	rs11465804	1.3×10^−25^	T/G	1.9 (1.65–2.01)	0.96/0.93	rs11209026	0.89^b^	0.97^b^	Burton *et al.*^3^
*IL23R*	1	67,702,526	rs11465804	1.3×10^−25^	T/G	1.9 (1.65–2.01)	0.96/0.93	rs11209032	0.03	1.00	Evans *et al.*^4^
*2p15*	2	62,551,472	rs10865331	1.2×10^−22^	A/G	1.3 (1.20–1.32)	0.43/0.38	rs10865331	1.00	1.00	Reveille *et al.*^5^
*21q22*	21	40,465,534	rs2836878	1.1×10^−16^	G/A	1.3 (1.19–1.32)	0.77/0.73	rs2836878	1.00	1.00	Reveille *et al.*^5^
*KIF21B-GPR25*	1	200,877,562	rs7554511	3.8×10^−16^	C/A	1.2 (1.18–1.30)	0.75/0.71	rs2297909	0.89	0.96	Evans *et al.*^4^
*GPR35*	2	241,569,692	rs3749171	5.0×10^−12^	T/C	1.2 (1.15–1.29)	0.21/0.18	rs4676410	0.86	1.00	Cortes *et al.*^11^
*IL6R*	1	154,426,970	rs2228145	1.9×10^−8^	A/C	1.1 (1.09–1.20)	0.62/0.59	rs4129267	0.97	1.00	Cortes *et al.*^11^
*ANTXR2*	4	80,949,829	rs4333130	5.3×10^−8^	T/C	1.1 (1.09–1.20)	0.66/0.63	rs4333130	1.00	1.00	Evans *et al.*^4^
*IL1R1*	2	102,663,628	rs2310173	2.3×10^−7^	T/G	1.1 (1.08–1.18)	0.50/0.47	rs4851529	0.48	0.89	Cortes *et al.*^11^
*IL1R1*	2	102,663,628	rs2310173	2.3×10^−7^	T/G	1.1 (1.08–1.18)	0.50/0.47	rs2192752	0.02	0.23	Cortes *et al.*^11^
*FCGR2A*	1	161,479,745	rs1801274	4.9×10^−6^	A/G	1.1 (1.06–1.16)	0.50/0.47	rs1801274	1.00	1.00	Cortes *et al.*^11^
*NOS2*	17	26,096,597	rs2297518	2.7×10^−6^	A/G	1.1 (1.08–1.21)	0.21/0.19	rs2297518	1.00	1.00	Cortes *et al.*^11^

Abbreviations: AS, ankylosing spondylitis; CI, confidence interval; Cs, chromosome; OR, odds ratio; Prot, protective; Pos, position; RAF, risk allele frequency; SNP, single-nucleotide polymorphism.

Linkage disequilibrium calculated from 1000 Genomes data via the SNAP Browser from the Broad Institute.

a Human Genome Build 19.

b Calculated from this data set.

**Table 2:** Novel AS associations

*Chr*	*SNP*	*Position*^a^	*Risk allele*	*Prot allele*	*Cases RAF*	*Cont RAF*	*OR (95% CI)*	P*-value*	*Gene/nearby gene*
*Genome-wide-significant association*									
6	rs6908425	20,728,731	C	T	0.81	0.78	1.2 (1.11–1.25)	1.8×10^−8^	*CDKAL1*
									
*Suggestive association*									
22	rs6007594	45,728,370	G	A	0.77	0.74	1.2 (1.10–1.22)	5.9×10^−8^	*FAM118A*
7	rs1456896	50,304,461	T	C	0.69	0.66	1.1 (1.08–1.20)	1.9×10^−7^	*C7orf72*
4	rs11555334	38,880,046	T	C	0.75	0.72	1.1 (1.08–1.20)	1.4×10^−6^	*FAM114A1*
6	rs141744967	36,270,205	T	C	0.0044	0.0017	2.6 (1.77–3.89)	1.5×10^−6^	*PNPLA1*

Abbreviations: AS, ankylosing spondylitis; Chr, chromosome; CI, confidence interval; Cont, control; OR, odds ratio; Prot, protective; RAF, risk allele frequency.

a Human Genome build 19.

**Table 3:** Secondary associations observed at loci associated with AS at genome-wide significance in the current study

*Chr*	*SNP*	*Position*^a^	*Gene*	P*-value*	*Risk/non-risk*	*RAF case/Con*	*OR (95% CI)*	*Conditional SNP*
5	rs2549794	96,244,549	*ERAP2*	2.9×10^−14^	C/T	0.44/0.43	1.22 (1.16–1.28)	rs30187
5	rs10050860	96,122,210	*ERAP1*	1.6×10^−9^	C/T	0.82/0.77	1.21 (1.14–1.29)	rs30187/rs2549794
1	rs10889677	67,725,120	*IL23R*	9.7×10^−9^	A/C	0.69/0.65	1.15 (1.10–1.21)	rs11465804
4	rs11096955	38,776,107	*TLR10*	3.5×10^−4^	G/T	0.32/0.30	1.09 (1.04–1.15)	rs11555334

Abbreviations: OR, odds ratio; Chr, chromosome; CI, confidence interval; RAF, risk allele frequency; SNP, single-nucleotide polymorphism.

a Human Genome build 19.

## Appendix

**Membership of Wellcome Trust Case Control Consortium 2 (WTCCC2)**

Management Committee

Peter Donnelly (Chair)^1,2^, Ines Barroso (Deputy Chair)^3^, Jenefer M Blackwell^4,5^, Elvira Bramon^6^, Matthew A Brown^7^, Juan P Casas^8^, Aiden Corvin^9^, Panos Deloukas^3^, Audrey Duncanson^10^, Janusz Jankowski^11^, Hugh S Markus^12^, Christopher G Mathew^13^, Colin NA Palmer^14^, Robert Plomin^15^, Anna Rautanen^1^, Stephen J Sawcer^16^, Richard C Trembath^13^, Ananth C Viswanathan^17^, Nicholas W Wood^18^

Data and Analysis Group

Chris CA Spencer^1^, Gavin Band^1^, Céline Bellenguez^1^, Colin Freeman^1^, Garrett Hellenthal^1^, Eleni Giannoulatou^1^, Matti Pirinen^1^, Richard Pearson^1^, Amy Strange^1^, Zhan Su^1^, Damjan Vukcevic^1^, Peter Donnelly^1,2^

DNA, Genotyping, Data QC and Informatics Group

Cordelia Langford^3^, Sarah E Hunt^3^, Sarah Edkins^3^, Rhian Gwilliam^3^, Hannah Blackburn^3^, Suzannah J Bumpstead^3^, Serge Dronov^3^, Matthew Gillman^3^, Emma Gray^3^, Naomi Hammond^3^, Alagurevathi Jayakumar^3^, Owen T McCann^3^, Jennifer Liddle^3^, Simon C Potter^3^, Radhi Ravindrarajah^3^, Michelle Ricketts^3^, Matthew Waller^3^, Paul Weston^3^, Sara Widaa^3^, Pamela Whittaker^3^, Ines Barroso^3^, Panos Deloukas^3^.

Publications Committee

Christopher G Mathew (Chair)^13^, Jenefer M Blackwell^4,5^, Matthew A Brown^7^, Aiden Corvin^9^, Chris CA Spencer^1^

^1^Wellcome Trust Centre for Human Genetics, University of Oxford, Roosevelt Drive, Oxford OX3 7BN, UK; ^2^Dept Statistics, University of Oxford, Oxford OX1 3TG, UK; ^3^Wellcome Trust Sanger Institute, Wellcome Trust Genome Campus, Hinxton, Cambridge CB10 1SA, UK; ^4^Telethon Institute for Child Health Research, Centre for Child Health Research, University of Western Australia, 100 Roberts Road, Subiaco, Western Australia 6008, Australia; ^5^Cambridge Institute for Medical Research, University of Cambridge School of Clinical Medicine, Cambridge CB2 0XY, UK; ^6^Department of Psychosis Studies, NIHR Biomedical Research Centre for Mental Health at the Institute of Psychiatry, King’s College London and The South London and Maudsley NHS Foundation Trust, Denmark Hill, London SE5 8AF, UK; ^7^University of Queensland Diamantina Institute, Brisbane, Queensland, Australia; ^8^Dept Epidemiology and Population Health, London School of Hygiene and Tropical Medicine, London WC1E 7HT and Dept Epidemiology and Public Health, University College London WC1E 6BT, UK; ^9^Neuropsychiatric Genetics Research Group, Institute of Molecular Medicine, Trinity College Dublin, Dublin 2, Eire; ^10^Molecular and Physiological Sciences, The Wellcome Trust, London NW1 2BE, UK; ^11^Department of Oncology, Old Road Campus, University of Oxford, Oxford OX3 7DQ, UK, Digestive Diseases Centre, Leicester Royal Infirmary, Leicester LE7 7HH, UK and Centre for Digestive Diseases, Queen Mary University of London, London E1 2AD, UK; ^12^Clinical Neurosciences, St George’s University of London, London, SW17 0RE, UK; ^13^King’s College London Dept Medical and Molecular Genetics, King’s Heath Partners, Guy’s Hospital, London SE1 9RT, UK; ^14^Biomedical Research Centre, Ninewells Hospital and Medical School, Dundee DD1 9SY, UK; ^15^King’s College, London Social, Genetic and Developmental Psychiatry Centre, Institute of Psychiatry, Denmark Hill, London SE5 8AF, UK; ^16^University of Cambridge Dept Clinical Neurosciences, Addenbrooke’s Hospital, Cambridge CB2 0QQ, UK; ^17^NIHR Biomedical Research Centre for Ophthalmology, Moorfields Eye Hospital NHS Foundation Trust and UCL Institute of Ophthalmology, London EC1V 2PD, UK; ^18^Dept Molecular Neuroscience, Institute of Neurology, Queen Square, London WC1N 3BG, UK.

**Membership of Australasian Osteoporosis Genetics Consortium (AOGC)**

Eugene McCloskey^1^, John Eisman^2^, Graeme Jones^3^, Geoff Nicholson^4^, Richard Eastell^5^, Philip Sambrook^6^ (deceased), Richard Prince^7^, Elaine Dennison^8^, Ian Reid^9^, John Wark^10^.

^1^Academic Unit of Bone Metabolism, University of Sheffield, UK; ^2^Garvan Institute of Medical Research, University of New South Wales, Australia; ^3^Menzies Research Institute, University of Tasmania, Australia; ^4^Rural Clinical School, University of Queensland, Australia; ^5^Academic Unit of Bone Metabolism, Metabolic Bone Centre, University of Sheffield, UK; ^6^Kolling Institute, Royal North Shore Hospital, University of Sydney, Australia; ^7^School of Medicine and Pharmacology, University of Western Australia, Australia; ^8^Medical Research Council Lifecourse Epidemiology Unit, University of Southampton, UK; ^9^Department of Medicine, University of Auckland, New Zealand; ^10^Department of Medicine, University of Melbourne, Australia.
