# Supplementary material for: Exome-wide study of ankylosing spondylitis demonstrates additional shared genetic background with inflammatory bowel disease
Source: NPJ Genom Med. 2016 May 4;1:16008–. doi: 10.1038/npjgenmed.2016.8 (PMC5685324; doi:10.1038/npjgenmed.2016.8)
Supplement: Supplementary Table 1 [file npjgenmed20168-s1.doc]

Supplementary Table 1: CDKAL1 associations and linkage disequilibrium with obesity and diabetes CDKAL1 associations

| AS SNP | Alternate phenotype SNP | r^2^ | D’ | Alternate phenotype | Reference |
| --- | --- | --- | --- | --- | --- |
| rs6908425 | rs2206734 | 0.03 | 1.00 | Body mass index | Okada *et al.* Nat Genet. 2012 Feb 19;44(3):302-6 |
| rs6908425 | rs7747752 | 0.04 | 1.00 | Glycated Hb levels | Ryu *et al.* Hum Mutat. 2012 Apr;33(4):655-9 |
| rs6908425 | rs7754840 | 0.09 | 1.00 | Type 2 DM | Wu *et al.* Diabetes. 2008 Oct; 57(10): 2834–2842 |
| rs6908425 | rs7756992 | 0.02 | 0.60 | Type 2 DM | Wu *et al.* Diabetes. 2008 Oct; 57(10): 2834–2842 |
| rs6908425 | rs7766070 | 0.03 | 0.60 | Type 2 DM | Perry *et al.* PLoS Genet. 2012;8(5):e1002741 |
| rs6908425 | rs9465871 | 0.03 | 1.00 | Type 2 DM | Wu *et al.* Diabetes. 2008 Oct; 57(10): 2834–2842 |
| rs6908425 | rs10946398 | 0.09 | 1.00 | Type 2 DM | Wu *et al.* Diabetes. 2008 Oct; 57(10): 2834–2842 |

Linkage disequilibrium sourced from the Broad SNAP browser with CEU 1000 genomes pilot 1 data; DM diabetes mellitus; Hb haemoglobin
