# Supplementary material for: Exome-wide study of ankylosing spondylitis demonstrates additional shared genetic background with inflammatory bowel disease
Source: NPJ Genom Med. 2016 May 4;1:16008–. doi: 10.1038/npjgenmed.2016.8 (PMC5685324; doi:10.1038/npjgenmed.2016.8)
Supplement: Supplementary Table 2 [file npjgenmed20168-s2.doc]

Supplementary Table 2: Functional annotation of novel and secondary variants

| SNP | Position* | In/near gene | Functional annotation |
| --- | --- | --- | --- |
| rs6908425 | 20,728,731 | *CDKAL1* | intron |
| rs6007594 | 45,728,370 | *FAM118A* | missense variant |
| rs1456896 | 50,304,461 | *C7orf72* | intergenic |
| rs11555334 | 38,880,046 | *FAM114A1* | missense splice region variant |
| rs141744967 | 36,270,205 | *PNPLA1* | missense variant |
| rs2549794 | 96,244,549 | *ERAP2* | tags missence mutation variant |
| rs10050860 | 96,122,210 | *ERAP1* | missense variant |
| rs10889677 | 67,725,120 | *IL23R* | 3’ untranslated region variant |
| rs11096955 | 38,776,107 | *TLR10* | missence variant |

*HG19
