# Supplementary figures and images for: Exome-wide study of ankylosing spondylitis demonstrates additional shared genetic background with inflammatory bowel disease
Source: NPJ Genom Med. 2016 May 4;1:16008–. doi: 10.1038/npjgenmed.2016.8 (PMC5685324; doi:10.1038/npjgenmed.2016.8)

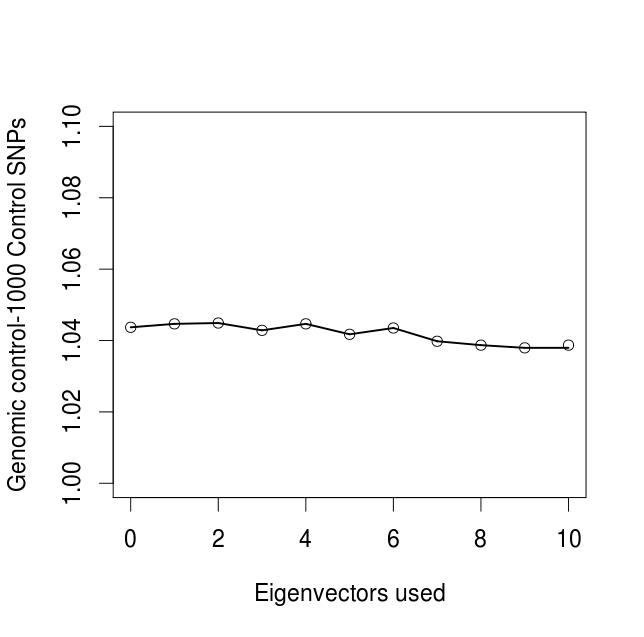

Supplement: Supplementary Figure 1 [file npjgenmed20168-s3.jpg]

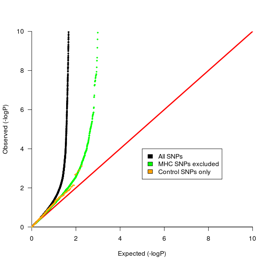

Supplement: Supplementary Figure 2 [file npjgenmed20168-s4.png]

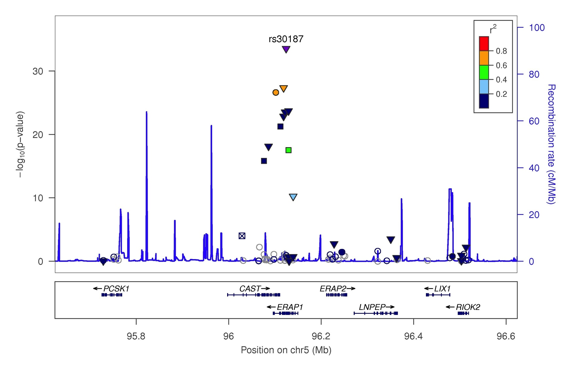

Supplement: Supplementary Figure 3 [file npjgenmed20168-s5.png]

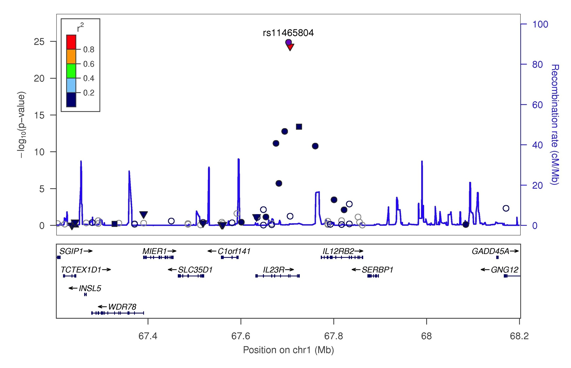

Supplement: Supplementary Figure 4 [file npjgenmed20168-s6.png]

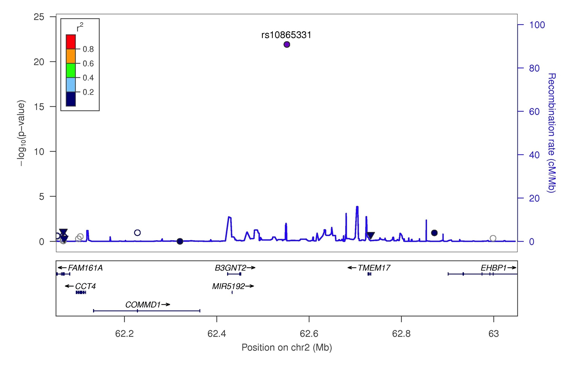

Supplement: Supplementary Figure 5 [file npjgenmed20168-s7.png]

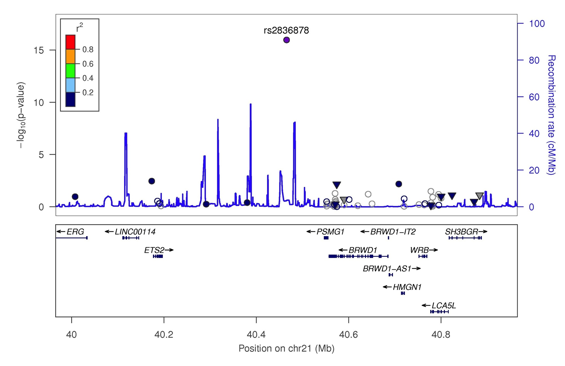

Supplement: Supplementary Figure 6 [file npjgenmed20168-s8.png]

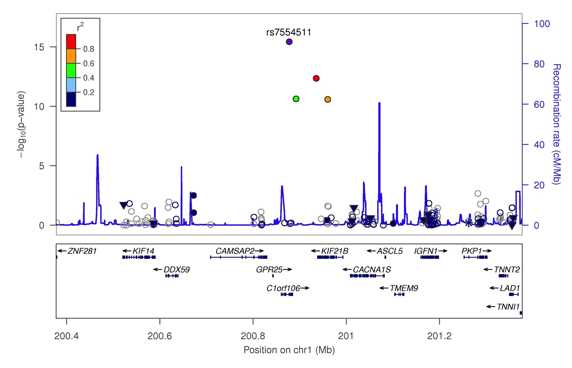

Supplement: Supplementary Figure 7 [file npjgenmed20168-s9.png]

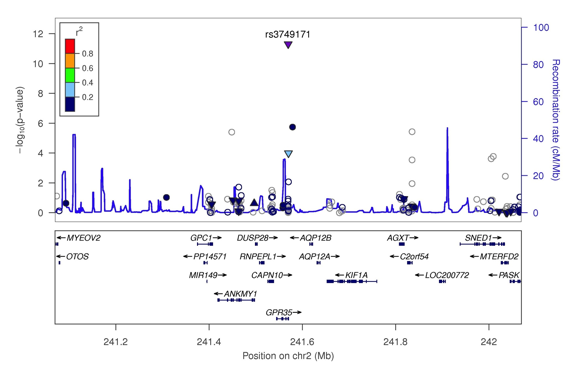

Supplement: Supplementary Figure 8 [file npjgenmed20168-s10.png]

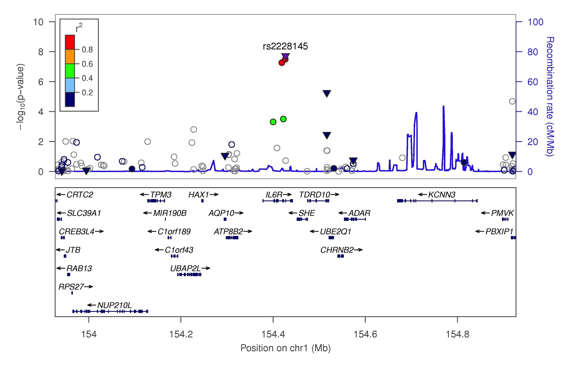

Supplement: Supplementary Figure 9 [file npjgenmed20168-s11.png]

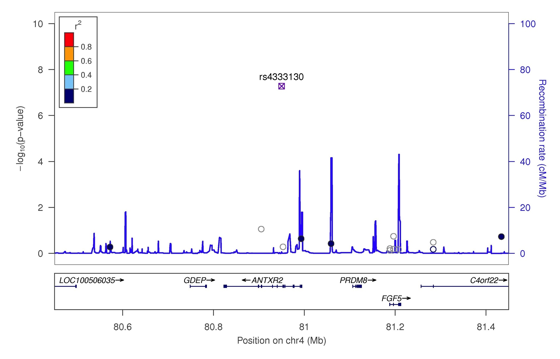

Supplement: Supplementary Figure 10 [file npjgenmed20168-s12.png]

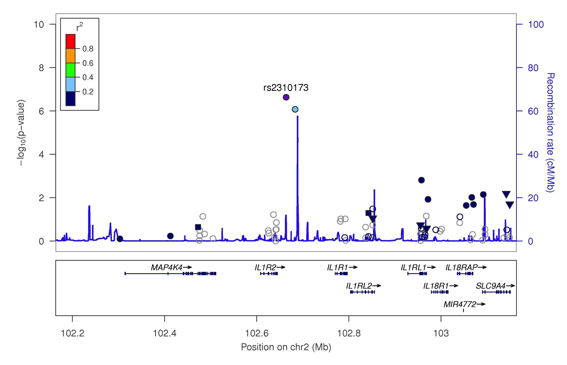

Supplement: Supplementary Figure 11 [file npjgenmed20168-s13.png]

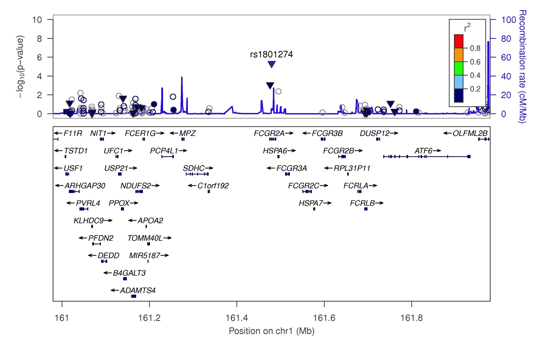

Supplement: Supplementary Figure 12 [file npjgenmed20168-s14.png]

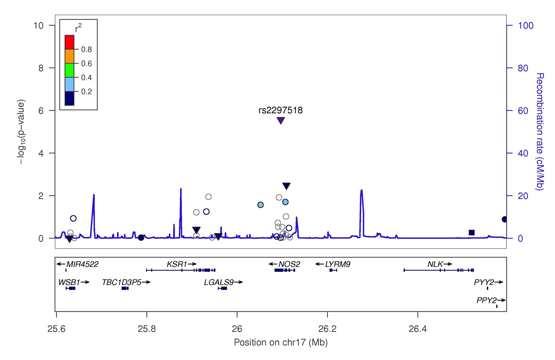

Supplement: Supplementary Figure 13 [file npjgenmed20168-s15.png]

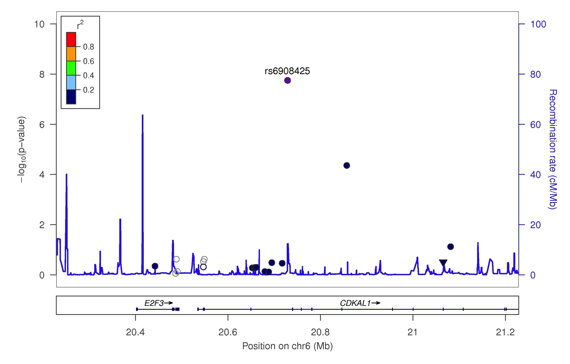

Supplement: Supplementary Figure 14 [file npjgenmed20168-s16.png]

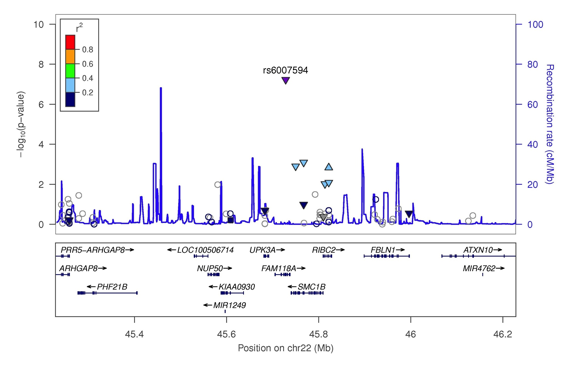

Supplement: Supplementary Figure 15 [file npjgenmed20168-s17.png]

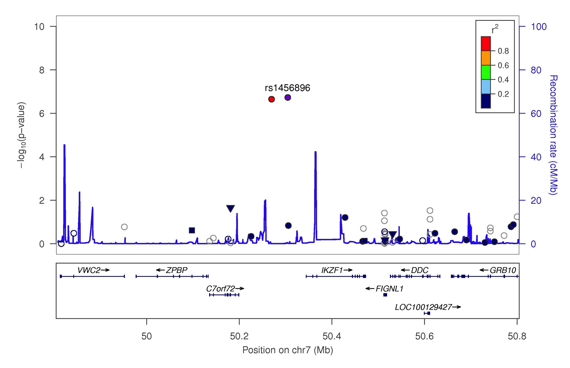

Supplement: Supplementary Figure 16 [file npjgenmed20168-s18.png]

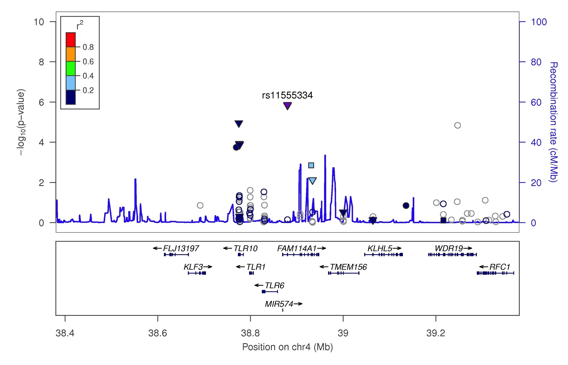

Supplement: Supplementary Figure 17 [file npjgenmed20168-s19.png]

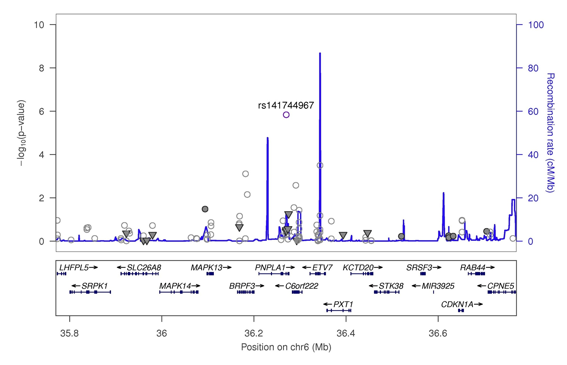

Supplement: Supplementary Figure 18 [file npjgenmed20168-s20.png]

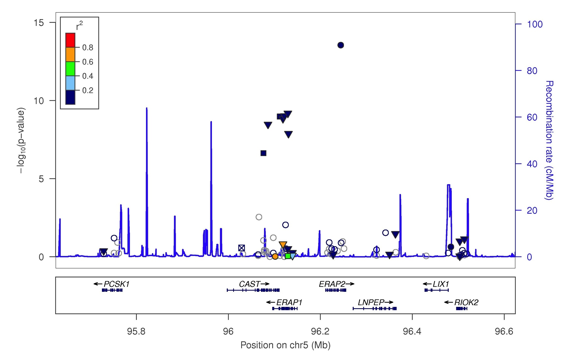

Supplement: Supplementary Figure 19 [file npjgenmed20168-s21.png]

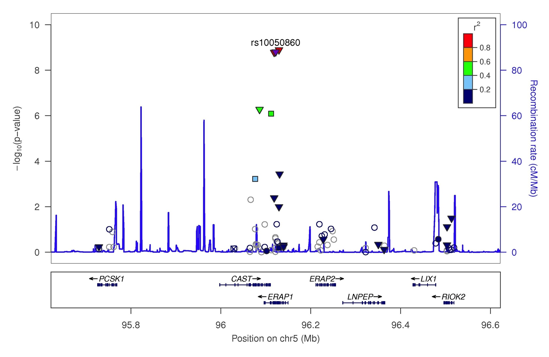

Supplement: Supplementary Figure 20 [file npjgenmed20168-s22.png]

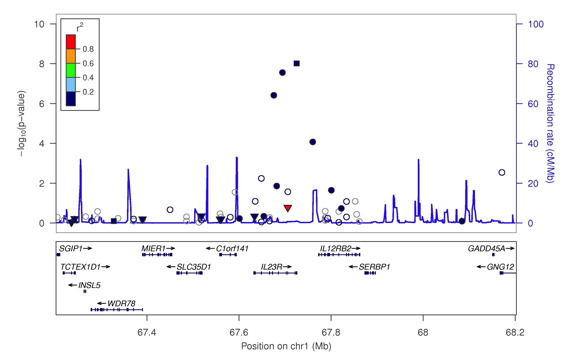

Supplement: Supplementary Figure 21 [file npjgenmed20168-s23.png]

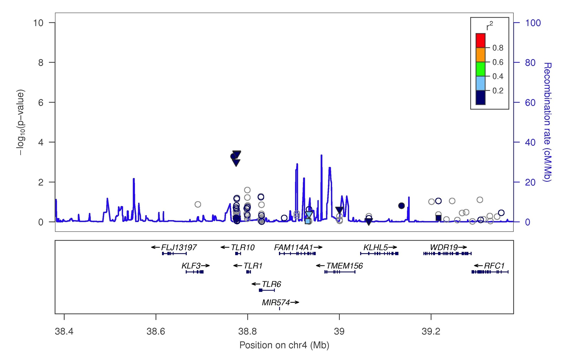

Supplement: Supplementary Figure 22 [file npjgenmed20168-s24.png]

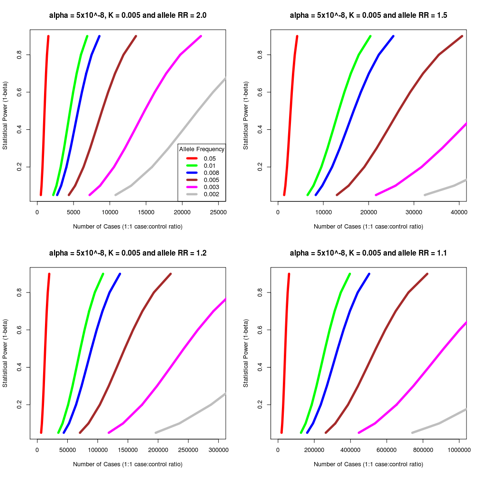

Supplement: Supplementary Figure 23 [file npjgenmed20168-s25.png]

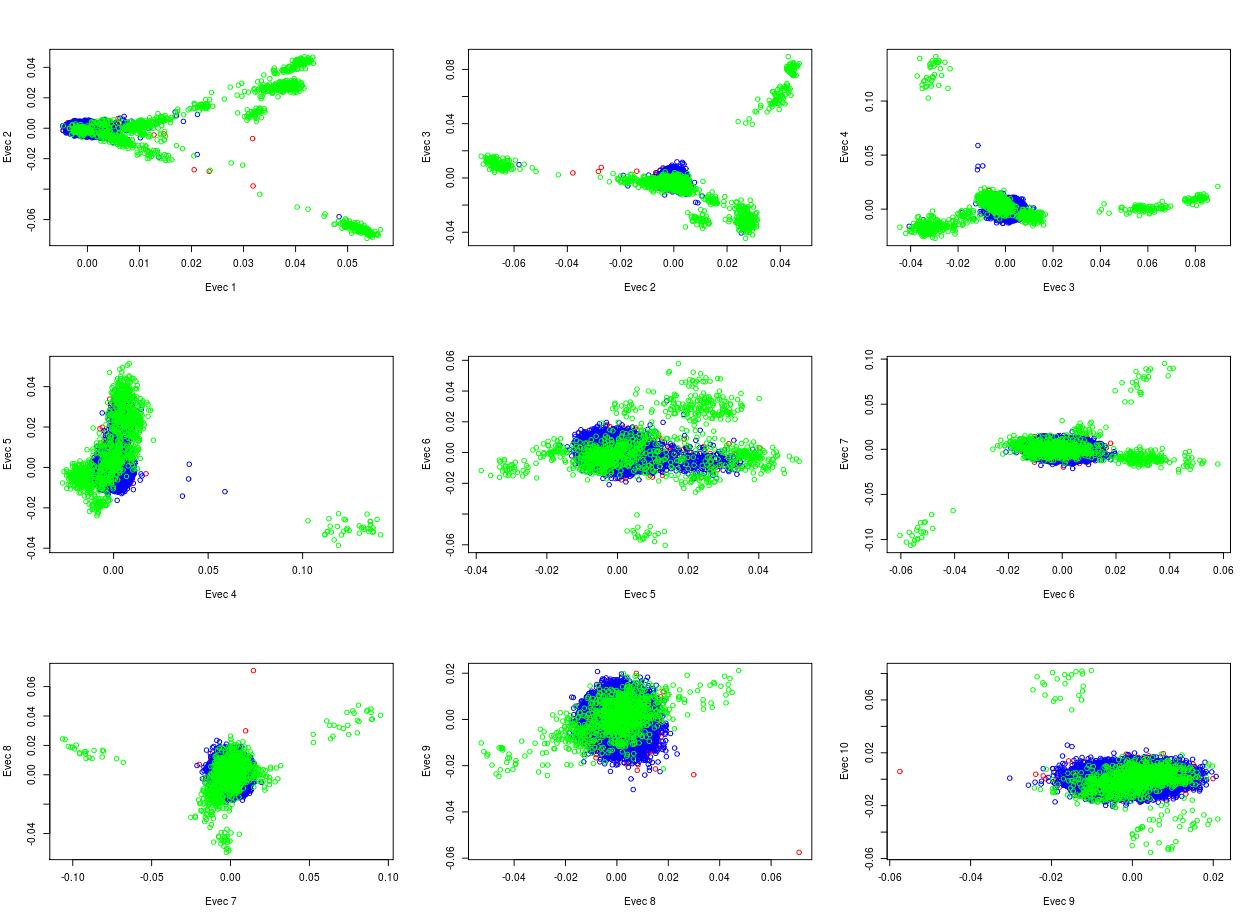

Supplement: Supplementary Figure 24 [file npjgenmed20168-s26.jpg]

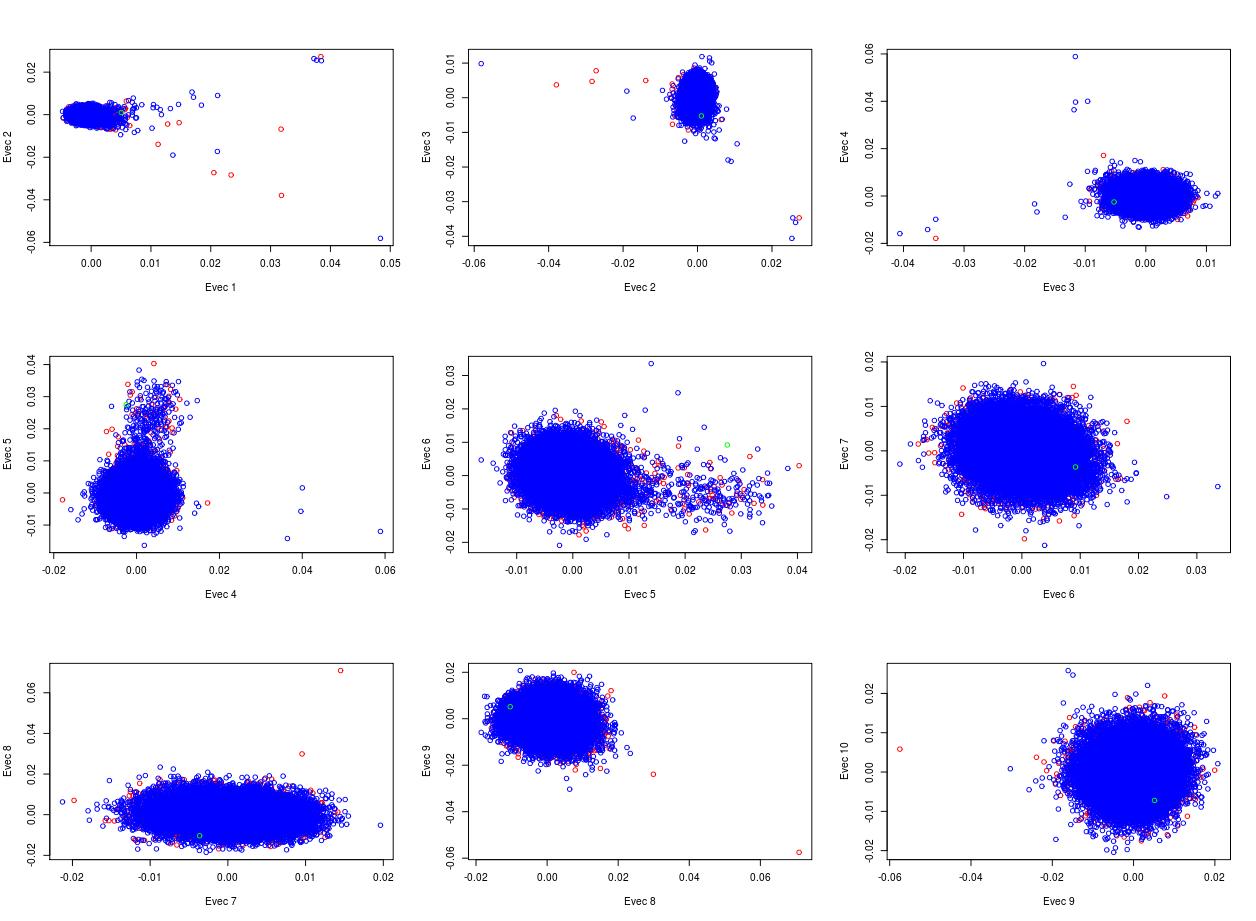

Supplement: Supplementary Figure 25 [file npjgenmed20168-s27.jpg]
