# Supplementary material for: Exome-wide study of ankylosing spondylitis demonstrates additional shared genetic background with inflammatory bowel disease
Source: NPJ Genom Med. 2016 May 4;1:16008–. doi: 10.1038/npjgenmed.2016.8 (PMC5685324; doi:10.1038/npjgenmed.2016.8)
Supplement: Supplementary Information [file npjgenmed20168-s28.doc]

Supplementary Figure 1: Scree plot of genomic control-1000 of control SNPs versus number of eigenvectors used

Supplementary Figure 2: Quantile-quantile plot for experiment

Supplementary Figure 3: Local genome plot of ERAP1 showing association at rs30187

Supplementary Figure 4: Local genome plot of IL23R showing association at rs11465804

Supplementary Figure 5: Local genome plot of intergenic region chromosome 2p15 showing association at rs10865331

Supplementary Figure 6: Local genome plot of intergenic region chromosome 21q22 showing association at rs2836878

Supplementary Figure 7: Local genome plot of GPR25-KIF21B showing association at rs7554511

Supplementary Figure 8: Local genome plot of GPR35 showing association at rs3749171

Supplementary Figure 9: Local genome plot of IL6R showing association at rs2228145

Supplementary Figure 10: Local genome plot of ANTXR2 showing association at rs4333130

Supplementary Figure 11: Local genome plot of IL1R1-IL1R2 showing association at rs2310173

Supplementary Figure 12: Local genome plot of FCGR2A showing association at rs1801274

Supplementary Figure 13: Local genome plot of NOS2 showing association at rs2297518

Supplementary Figure 14: Local genome plot of CDKAL1 showing association at rs6908425

Supplementary Figure 15: Local genome plot of FAM118A showing association at rs6007594

Supplementary Figure 16: Local genome plot of C7orf72 showing association at rs1456896

Supplementary Figure 17: Local genome plot of FAM114A1 showing association at rs11555334

Supplementary Figure 18: Local genome plot of PNPLA1 showing association at rs141744967

Supplementary Figure 19: Local genome plot of ERAP1-ERAP2 showing at rs2549794 after conditioning on rs30187

Supplementary Figure 20: Local genome plot of ERAP1 showing association at rs10050860 after conditioning on rs30187 and rs2549794

Supplementary Figure 21: Local genome plot of IL23R showing association at rs10889677 after conditioning on rs11465804

Supplementary Figure 22: Local genome plot of FAM114A1 showing association at rs11096955 after conditioning on rs11555334

Supplementary Figure 23 This figure shows the relationship between statistical power, case number, effect size of allele (RR – relative risk) and allele frequency. K indicates the assumed population prevalence (0.5%), alpha indicates required significance level, set in these examples to the genomewide significance level (5 ×10^-8^.

Supplementary Figure 24 Post quality control samples with Hap Map samples included. Hapmap samples are shown in green, case samples shown in red and controls shown in blue. Evec indicates ‘eigenvector’ n value, and as such the scale is relative ad arbitrary.

Supplementary Figure 25 Post quality control samples with Hap Map samples excluded. Case samples shown in red and controls shown in blue. Evec indicates ‘eigenvector’ n value, and as such the scale is relative ad arbitrary.
